# Role of TL1A in Inflammatory Autoimmune Diseases: A Comprehensive Review

**DOI:** 10.3389/fimmu.2022.891328

**Published:** 2022-07-14

**Authors:** Wang-Dong Xu, Rong Li, An-Fang Huang

**Affiliations:** ^1^ Department of Evidence-Based Medicine, Southwest Medical University, Luzhou, China; ^2^ Department of Rheumatology and Immunology, Affiliated Hospital of Southwest Medical University, Luzhou, China

**Keywords:** TL1A, inflammatory autoimmune disease, immune response, DR3, TNFSF15

## Abstract

TL1A, also called TNFSF15, is a member of tumor necrosis factor family. It is expressed in different immune cell, such as monocyte, macrophage, dendritic cell, T cell and non-immune cell, for example, synovial fibroblast, endothelial cell. TL1A competitively binds to death receptor 3 or decoy receptor 3, providing stimulatory signal for downstream signaling pathways, and then regulates proliferation, activation, apoptosis of and cytokine, chemokine production in effector cells. Recent findings showed that TL1A was abnormally expressed in autoimmune diseases, including rheumatoid arthritis, inflammatory bowel disease, psoriasis, primary biliary cirrhosis, systemic lupus erythematosus and ankylosing spondylitis. *In vivo* and *in vitro* studies further demonstrated that TL1A was involved in development and pathogenesis of these diseases. In this study, we comprehensively discussed the complex immunological function of TL1A and focused on recent findings of the pleiotropic activity conducted by TL1A in inflammatory autoimmune disease. Finish of the study will provide new ideas for developing therapeutic strategies for these diseases by targeting TL1A.

## Introduction

Tumor necrosis factor (TNF)-like cytokine 1A (TL1A), a member of the TNF superfamily, was first identified in 2002 (1). The tnfsf15 gene encoding TL1A is located at chromosome 9q32 in human and chromosome 4 in mouse. TL1A is a type 2 transmembrane protein that self-assembles into stable trimers by interacting with TNF homology domain (THD). It is mainly expressed as the membrane-bound form, and it forms stable trimers. The soluble TL1A (sTL1A) was produced by alternative splicing or TNF-α-converting enzyme (TACE) cleavage (1, 2). TL1A is constitutively expressed in endothelial cell, and it is up-regulated in response to tumor necrosis factor-α (TNF-α) stimulation. Expression of TL1A in dendritic cell (DC) and macrophage is increased when the cells were triggered by toll-like receptors 4 (TLR4), TLR11 or Fc region of IgG (FcγR) (3–5). Mitogen-activated protein kinases (MAPKs), nuclear factor kappa-light-chainenhancer of activated B cells (NF-κB), caspase-8 signaling pathways regulate immune response, ranging from apoptosis to autoimmunity (6, 7). TL1A binds to its receptor death receptor 3 (DR3), activates downstream signalings, and then participates in innate and adaptive immune homeostasis. It has been shown that sTL1A can be detected in serum and body fluids of patients with T cell-mediated inflammatory autoimmune diseases, such as rheumatoid arthritis (RA), psoriatic arthritis (PsA), and ankylosing spondylitis (AS). TL1A also plays important roles in the pathogenesis of these diseases (8). In recent years, TL1A, as an important mediator of inflammation, has attracted much attention because anti-TL1A antibody treatment may be a promising therapeutic approach in inflammatory disorders (9). Here, this study summarized information about the molecular mechanism of TL1A and discussed role of TL1A in immune cells, especially focused on association between TL1A and inflammatory autoimmune diseases.

## Receptors and Signaling Pathways of TL1A

**Figure 1:**
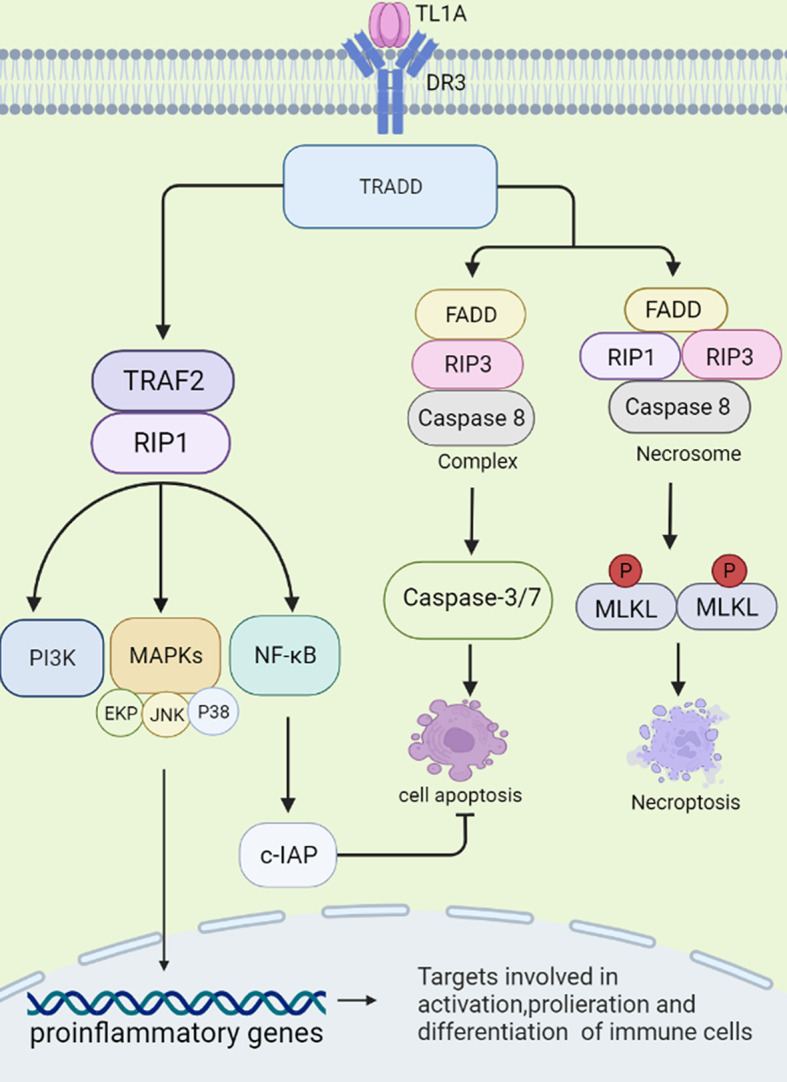
Signal transduction initiated by TL1A/DR3. TL1A binds to the receptor DR3 and activates the TRADD pathway. The complex exerts pro-inflammatory effects by regulating downstream pathways, such as TRAF2, RIP1, PI3K, MAPKs, NF-κB, and then regulates cytokine, chemokine secretion. TL1A/DR3 is involved in promoting apoptosis and necroptotic cell death through FADD, RIP3, Caspase-8/-3/-7 pathways. NF-κB can activate c-IAP protein, which can negatively regulate apoptosis. TRADD, TNFR-associated death domain protein; TRAF2, TNFR-associated factor 2; RIP1, receptor-interacting protein 1; PI3K, phosphatidylinositol 3-kinase; MAPKs, mitogen-activated protein kinases; NF-κB, nuclear factor kappa B; FADD, fas-associated death domain; RIP3, receptor-interacting protein 3; c-IAP, cellular inhibitor of apoptosis proteins.

DR3 is a type 1 membrane protein and contains four cysteine residues, two potential N-linked glycosylation sites, a transmembrane domain and a cytoplasmic domain with a death domain (DD). Osteoblasts are capable of producing transmembrane and soluble forms of DR3. In resting T cells, DR3 is expressed in a soluble form that protects the cells from apoptosis. Activated T cells express transmembrane DR3. It activates receptors, leading to apoptosis or activation of transcription factors such as NF-κB (10–12). sTL1A is sufficient to bind DR3 (membrane-bound form) to activate downstream signaling cascades, and therefore modulates immune response and inflammation (1, 13). Two different signaling pathways could be triggered by sTL1A/DR3 interaction, causing inflammation and apoptosis, respectively (10). First, death domain of DR3 combined with adapter protein TNFR-associated death domain protein (TRADD) in the cytoplasm, and then recruited TNFR-associated factor 2 (TRAF2) and receptor-interacting protein 1 (RIP1) (8, 14). These complexes activated MAPKs (ERK, p38, and JNK), NF-κB, and the effector kinases PI3K signaling (3). Finally, the activated signalings regulated expression of pro-inflammatory genes and participated in occurrence of immune related diseases. TRADD binds to Fas-associated death domain (FADD) and RIP3, and then activates cysteinyl aspartate specific proteinase-8 (caspase-8) to form complexes, which further activate downstream caspase pathways (for example, caspase-3 and -7) and induce apoptotic cell death. Combination of FADD, RIP3, RIP1 and the downstream effector molecule mixed lineage kinase domain-like protein (MLKL) forms a cytosolic “necrosome” complex after phosphorylation when caspase-8 activity is blocked. Then, MLKL oligomerizes to the cell membrane, and causes necroptotic cell death, a form of cell death with intense inflammation (15, 16). In this process, NF-κB is proved to induce activation of cellular inhibitor of apoptosis proteins (c-IAP), which mediates negative feedback regulation and inhibition of cell apoptosis (15). In fact, more evidence has shown that connection of sTL1A to DR3 preferentially induces pro-inflammatory pathways rather than apoptosis in lymphocytes (**Figure 1**) (17).

Interestingly, soluble decoy receptor 3 (DcR3), is another receptor for TL1A, which lacks a cytoplasmic domain to regulate cell function by ‘decoy’ and ‘non-decoy’ action (18). When DcR3 competitively binds to sTL1A, combination of sTL1A and DR3 may be destroyed, and the complex results in less lymphocyte activation and pro-inflammatory cytokine production and prevents apoptosis (19). Thus, if targeting DcR3 is applied to clinic usage, inhibiting expression of DcR3 may inhibit tumor growth and promote tumor apoptosis, whereas increasing expression of DcR3 is expected to become a promising therapeutic method in autoimmune diseases (18). Interestingly, DcR3 is able to bind other ligands, such as FasL and LIGHT. FasL is a pro-inflammatory and pro-apoptotic cytokine and LIGHT is involved in apoptosis and inflammation. DcR3 acts as an immunomodulator to inhibit apoptosis, reduce inflammation and prevent tissue damage by neutralizing LIGHT and FasL (20). Therefore, sTL1A/DR3 binding may promote cytokine secretion, lymphocyte proliferation and cell apoptosis, whereas DcR3 may abolish effects of sTL1A/DR3.

TL1A also binds to TNFR2 to produce pro-inflammatory components, such as IL-6, reactive oxygen species (ROS), and then impairs mitochondrial membrane of fibroblast-like synoviocytes (FLSs), leading to mitochondrial dysfunction of FLSs (21). Moreover, TL1A/TNFR2 axis increased migration and attachment of RA-FLSs to cartilage and bone *via* Indian Hedgehog signaling pathway, resulting in inflammatory response in arthritis (22).

## Biologic Functions of TL1A

TL1A can be generated by umbilical vein endothelial cells (HUVEC), monocyte, macrophage, dendritic cell (DC), T cell, chondrocyte, and synovial fibroblast, which are sources of both soluble and membrane-bound forms of TL1A (23–25). TL1A participates in intrinsic immunity by affecting mononuclear phagocytes, DCs, natural killer cells (NK cells), and innate lymphoid cells (ILCs) to regulate the immune environment. sTL1A exerts multifarious effects on adaptive immune cells by binding DR3, which affects activation, proliferation, differentiation of different immune cells, such as helper T cell, regulatory T cells, B cells and production of cytokines.

### Effect of TL1A on Innate Immunity

Mononuclear phagocytes in the lamina propria of mouse and human intestine are capable to express TL1A. Macrophages overexpressing TL1A promoted secretion of TNF-α and IL-1β (26). Monocytes secreted TL1A in response to stimulation by immune complexes, leading to enhanced T cell responses (5, 27). In inflammatory bowel disease, TL1A is produced in the intestinal lamina propria of macrophages in patients with Crohn’s disease (23).

TL1A activated DCs and promoted migration of DCs, thereby exacerbating development of inflammatory diseases (28). The receptor for TL1A, DcR3, is able to directly regulate DCs differentiation and maturation, and then modulates T cells differentiation and function (29). TL1A binding to DR3 synergies with IL-12 and IL-18 to increase interferon-γ (IFN-γ) production in natural killer cells (NK cells) (30). TL1A enhances NK cells infiltration and anti-tumor response as well as NK cells cytotoxicity to target cells (31, 32). In the antigen-induced arthritis (AIA) mice model, DR3 treatment increased neutrophils proliferation, and released much neutrophil chemokine (C-X-C motif) Ligand 1 Protein (CXCL1), gelatinase matrix metalloproteinase 9 (MMP-9), driving early cartilage destruction (33).

**Figure 2:**
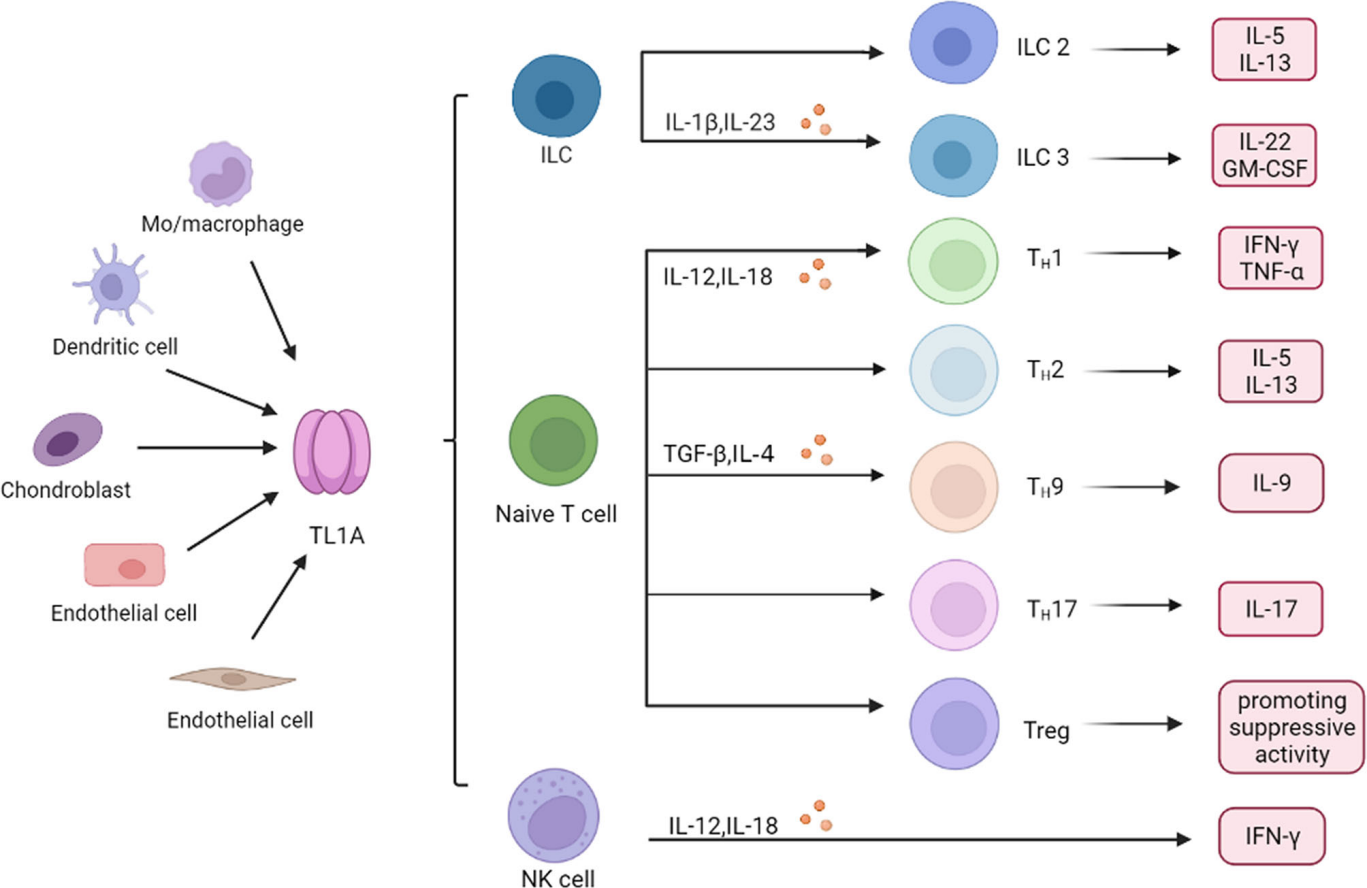
Role of TL1A in immune cells. Monocyte (Mo) and macrophage, endothelial cells, dendritic cell, chondrocyte, and synovial fibroblast produce TL1A, which regulates production of inflammatory cytokines and chemokines in different immune cells, including ILC, T cells and NK cells. ILC, innate lymphoid cells; NK cell, natural killer cell; Th, helper T cell; IL-1β, interleukin-1β; TNF-α, tumor necrosis factor-α; TGF-β, transforming growth factor-β; GM-CSF, granulocyte­macrophage colony stimulating factor; IFN-γ, interferon−γ.

As regulators of innate immunity, innate lymphoid cells (ILCs) have been shown to play a key role in mucosal homeostasis and inflammatory diseases. High DR3 expression was found in both human and mice ILCs, which increases cytokine production such as IL-25, IL-33 in response to TL1A stimulation (34, 35). Role of type 2 cells (ILC2) is dependent on the TL1A/DR3 axis, which promotes expansion, survival and function of ILC2 (34). TL1A and DR3 co-stimulates IL-5 and IL-13 production by ILC2, driving allergic pathology and participating in development of allergic lung inflammation (36). ILC3 regulates intestinal immunity, while activation of DR3 signaling pathway leads to ILC3 loss and promotes intestinal inflammation (37). TL1A can synergy with IL-23 and IL-1β to increase ILC3 proliferation and produce cytokines such as IL-22 and granulocyte macrophage colony stimulating factor (GM-CSF), suggesting a central role for TL1A in promoting the ILC3 immune barrier (38, 39). Collectively, TL1A binds to receptors and then regulates innate immune response (**Figure 2**).

### Significant Role of TL1A in Adaptive Immunity

TL1A synergies with IL-12 and IL-18 to induce secretion of IFN-γ and TNF-α, thereby boosting helper T cell 1 (Th1) immune response (30, 40). However, function of TL1A in the adaptive immune system is not limited to Th1 immune response. TL1A activated T cells, promoting IL-5 and IL-13 production in the intestinal mucosa, which are characteristic cytokines produced by helper T cell 2 (Th2) cells (30, 41). Blocking TL1A disrupted IL-13 secretion, suggesting that TL1A is critical in Th2 immune response (19). Interestingly, role of TL1A in helper T cell 17 (Th17) response appears dichotomous. TL1A has been reported to inhibit generation and polarization of Th17 cells in mice and human CD4+ T cells by binding to DR3 (42). By contrast, some studies showed that TL1A could augment Th17 differentiation through up-regulation of (retinoic acid-related orphan nuclear receptor C (RORc) expression (a Th17 lineage-specific transcription factor) and induce the proliferation of Th17 cells upon DR3 activation (8, 42, 43). The evidence indicated that TL1A aggravates inflammatory reactions through regulating Th17 pathway. It is known that TL1A also regulates generation of Th9 cells that are involved in the pathogenesis of IBD and allergic lung inflammatory disease (44, 45). TL1A up-regulated transforming growth factor-β (TGF-β) and IL-4 expression to stimulate Th9 differentiation and IL-9 secretion (44, 46). TL1A mediates Th9 differentiation through an IL-2 and STAT5-dependent mechanism (**Figure 2**) (45).

Compared to wild-type (WT) mice, a population of CD4+ T cells expressing IFN-γ was significantly decreased in TL1A gene deficient mice, while activation of the TL1A/DR3 pathway resulted in higher levels of Th1 cytokine production in SAMP1/YitFc (SAMP) mice and mitigated anti-inflammatory process during Chronic Ileitis (35, 42). Transgenic mice that were constitutively expressed TL1A in T cells or DCs, significantly developed IL-13-dependent inflammatory intestinal pathology, suggesting that TL1A is involved in the Th2 immune regulation (47). In transgenic mice with overexpression of TL1A, as well as in UC patients, there was enhanced Th9 cell differentiation, IL-9, TGF-β, and IL-4 expression (38). Similarly, in allergic lung disease mice model, binding of TL1A to DR3 could enhance differentiation and pathogenicity of Th9 cells. Exogenous TL1A suppresses the differentiation of naïve CD4+ T cells into Th17 cells, which is independent of STAT1 signaling and IL-2 signaling (48). TL1A, alone or in combination with IL-23, stimulated peripheral blood mononuclear cells (PBMCs) to produce IL-17 in autoimmune diseases, such as psoriasis vulgaris (PV) (49, 50), systemic sclerosis (SSc) (51), IBD (52). TL1A induced high levels of IL-22 in memory CD4+ T cells and committed Th17 cells through secretion of IL-9 (53). Moreover, DCs lacking TL1A had a reduced ability to induce Th17 differentiation and proliferation in experimental autoimmune encephalomyelitis (EAE) (26), and anti-TNF-α treatment inhibited effects of TL1A-mediated Th17 differentiation in RA (43).

Regulatory T cells (Treg) are a subset of CD4+ T cells, and maintain effector cells activation and proliferation. Activation of the TL1A/DR3 axis leads to Treg cells expansion and activation, and controls exuberant immune activation (**Figure 2**) (47, 54, 55). However, some studies showed that triggering DR3 in conventional effector CD4+ T cells and Treg cells enables TL1A to directly or indirectly attenuate the suppressive function of Treg cells (56). These inconsistent findings may suggest that the effect of TL1A on Treg cells might be highly dependent on the immune response environment regulated *in vivo* or *in vitro* (13, 57). However, more studies are needed to better clarify exact role of TL1A/DR3 in Treg cells.

Compared with T cells, there is limited evidence discussed effects of TL1A on B cells. A study revealed that plasma cells are the direct targets of TL1A, and TL1A promotes plasma cells survival and different antibodies production, which in turn enhance pathogenic antibodies production in CIA (collagen induced arthritis) mice (58). TL1A inhibits B cell proliferation and helps effector B cells to maintain immune homeostasis (59).

Therefore, TL1A is a pivotal mediator in adaptive immunity, including T and B cells.

## Association Between TL1A and Inflammatory Autoimmune Diseases

### Rheumatoid Arthritis

Rheumatoid arthritis (RA) is a chronic systemic autoimmune disease characterized by erosive arthritis. The main pathological features of RA are joint synovial hyperplasia, formation of pannus, bone destruction, and joint dysfunction (60). FLSs are major component of hyperplastic synovial tissue and involve in the inflammatory process and pathogenesis of RA (61). TL1A enhanced differentiation of Th17 cells induced by IL-6 and TGF-β stimulation through activation of RORc. The cytokine IL-17 produced by Th17 can prolong the survival of RA-FLSs and germinal center immune cells. FLSs then trigger cartilage degradation by producing inflammatory factors and matrix degradation molecules, damaging the joint and ultimately leading to RA (43, 60).

CIA is a model of human RA with similar histopathological and clinical features (62). After treatment with anti-TL1A antibody, total joint score was decreased and clinical inflammation of CIA mice was effectively alleviated, which was mediated by blocking the TL1A/DR3 signaling pathways (63). Similarly, compared to WT mice, TL1A gene knockout mice had improved clinical profiles for CIA (improved synovial hyperplasia and cartilage erosion) through reduction of inflammatory cells infiltration and reduced production of pathogenic antigens (58). *In vitro* experiments showed that in the presence of Receptor Activator of Nuclear Factor-κ B Ligand (RANK-L) and macrophage colony-stimulating factor (M-CSF), DR3-dependent TL1A promoted osteoclast formation in RA patients and mice model, suggesting that TL1A drives the bone pathology of RA (64).

TL1A levels in serum and synovial fluid were higher in RA patients when compared to that in healthy controls and correlated with expression of rheumatoid factor (RF), anti-cyclic citrullinated peptide (anti-CCP), as well as disease activity (**Table 1**) (63, 68, 69). RF and anti-CCP antibodies are promising disease markers for early diagnosis and prognosis of RA (63). Regarding genomics for TL1A gene, association of TL1A gene single-nucleotide polymorphism (SNP) (rs3810936 and rs7848647) with susceptibility to RA was discussed in Chinese population. TC, TT+TC genotypes of rs3810936, rs7848647 were negatively correlated with RA risk (**Table 2**). The CC genotype of TL1A rs7848647 polymorphism contributes to elevated TL1A levels and is associated with antibody production (RF expression) (65). Expression of genes related to proliferation of RA-FLSs, including spectrin repeat-containing nuclear envelope 1 (SYNE1), Fc receptor-like 2 (FCRL2), PYD (pyrin domain)-containing 1 (PYDC1), cell division cycle 45 homolog (CDC45), signal transducer and activator of transcription 5B (STAT5B), and interferon regulatory factor 4 (IRF4), was increased after TL1A stimulation (70). The progressive course of RA implicates several systems damage, especially the cardiovascular system. Atherosclerosis is the most common complication in RA patients (71). A study found that elevated serum levels of TL1A in RA patients were significantly associated with progression of atherosclerotic plaque height, which may increase the risk of atherosclerosis in RA patients (75).

**Table 1:** Expression of TL1A in inflammatory autoimmune diseases.

Disorder	Expression of TL1A	References
Rheumatoid arthritis	Serum,synovial fluid	(56-58)↑
Inflammatory bowel disease	Colonic tissues	(3, 65)↑
Psoriasis	Serum	(20, 40)↑
Primary biliary cirrhosis	Serum	(66)↑
Systemic lupus erythematosus	Serum	(41)↑
Ankylosing spondylitis	Serum	(67)↑

↑, increase.

**Table 2:** Relationship between *tnfsf15 gene* polymorphism and susceptibility to inflammatory autoimmune diseases.

Disease susceptibility	Ethnicity	Polymorphism	References
RA	Chinese Han population	rs3810936, rs7848647	(59)
IBD	British	rs6478109	(70)
	Chinese Han population	rs10114470	(71)
PsA	Hungarians	rs6478109	(72)
PBC	Chinese Han populations, Japanese	rs4979462	(66, 73)
SLE	Chinese Han population	rs3810936, rs7848647	(74)
AS	Chinese Han population	rs3810936	(75)

RA, rheumatoid arthritis; IBD, inflammatory bowel disease; PsA, psoriasis arthritis; PBC, Primary biliary cirrhosis; SLE, systemic lupus erythematosus; AS, ankylosing spondylitis.

### Inflammatory Bowel Disease

Inflammatory bowel disease (IBD), including ulcerative colitis (UC) and Crohn’s disease (CD), is a group of chronic inflammatory intestinal diseases, and the pathogenesis was associated with susceptible genes, dysregulated immune system, and environmental factors (76).

TL1A affects epithelial to mesenchymal transition (EMT) in IBD patients *via* the TGF-β/Smad3 pathway, causing colonic fibrosis and inflammatory responses (77). TL1A is capable of increasing the barrier permeability of TNF-α-induced Caco-2 cell and reducing function of tight junction protein (TJ) through myosin light chain kinase/p-myosin II regulatory light chain (MLCK/p-MLC) pathway and LPS-mediated myeloid differentiation factor 88/TNF receptor-associated factor-6 (MyD88/TRAF6) pathway, which further damage the intestinal mucosal barrier (78, 79). TL1A induces expression of pro-inflammatory cytokines such as TNF-α to regulate intestinal microenvironment and aggravates intestinal inflammation (40). For instance, dextran sodium sulfate (DSS)-induced colitis mice with TL1A overexpression had more severe intestinal inflammation and bacterial translocation, suggesting that TL1A promotes intestinal mucosal barrier disruption (80).

TL1A expression was elevated in colonic tissues of IBD patients compared to healthy controls and correlated with severity of inflammation, especially in CD patients (**Table 1**) (23, 77). The IBD protective allele A (rs6478109) was associated with increased tnfsf15 gene expression, indicating that IBD genetic susceptibility may relate to changes in TL1A expression (81). Rs10114470 polymorphism is considered to be a loci for IBD susceptibility in Chinese Han population (**Table 2**) (80). Furthermore, TL1A gene rs6478109 polymorphism is an independent factor of surgery, which affects the long-term efficacy of anti-TNF antibody therapy (72). Interestingly, anti-TL1A drug (PF-06480605) improved tissue inflammation and inhibited expression of fibrotic pathways, and reduced intestinal pathogens in UC patients (82). To date, this drug underwent preclinical safety and tolerability testing and is expected to be used as a clinical trial drug for treatment of IBD-related diseases. However, further studies with PF-06480605 in patients with other inflammatory diseases are needed to confirm the effects and to clarify the mechanism in inhibiting the diseases (9, 66).

### Psoriasis

Psoriasis is a common inflammatory skin disease characterized by thickened, scaly, red skin patches (73). TL1A is predominantly expressed in psoriatic lesions, particularly in infiltrating inflammatory cells, keratinocytes and vascular cells (27, 74). TL1A promotes production of IL-17, which induces granulocyte colony-stimulating factor (G-CSF) and chemokine ligand 20 (CCL20) and recruits large number of neutrophils to damaged joints, leading to early inflammation. IL-17 also promotes osteoclast formation, causing local bone damage that may contribute to development of psoriatic arthritis (83). Imiquimod (IMQ)-induced elevated TL1A expression at lesions in psoriasis-like mice, and elevated TL1A exacerbated the psoriasis phenotype through increasing the number of T cells, neutrophils, and DCs, and upregulating inflammatory cytokines IL-17 and IFN-γ. After treatment with anti-TL1A antibody, the lesions were effectively alleviated and histopathological changes were significantly reduced, suggesting that TL1A is involved in the pathogenesis of psoriasis (49). TL1A can synergy with IL-23 to stimulate IL-17 secretion in PBMCs from psoriasis vulgaris patients, thereby aggravating the disease (50).

TL1A and DR3 protein levels, as well as mRNA expression, were much higher in patients with psoriasis than in healthy controls, and both of them were decreased after treatment (**Table 1**) (50, 67). A study found that in Hungarians, TL1A gene rs6478109 polymorphism was associated with susceptibility to psoriasis. The haplotype AGTAA (rs3810936 (A)+rs6478108 (G)+rs6478109 (T)+rs7848647 (A)+rs7869487 (A) may have a protective effect in disease pathogenesis (**Table 2**) (84). Collectively, TL1A may play a role in development of psoriasis.

### Primary Biliary Cirrhosis

Primary biliary cirrhosis (PBC) is an autoimmune liver disease caused by biliary tract obstruction and cholestasis. TL1A is mainly expressed in biliary epithelial cells, vascular cells and infiltrating mononuclear cells of PBC liver (85). In mice model, TL1A may exacerbate liver fibrosis by recruiting macrophages and promoting secretion of pro-inflammatory cytokines (26). Compared with healthy controls, serum levels of TL1A were significantly elevated in PBC patients and decreased after ursodeoxycholic acid (UDCA) treatment (**Table 1**). TL1A gene rs4979462 polymorphism was a PBC susceptible polymorphism in Japanese and Chinese Han population (**Table 2**) (86, 87). The above results suggest that TL1A is associated with disease progression and may be involved in the pathogenesis of PBC.

### Other Systemic Inflammatory Diseases

TL1A has emerged as a key inflammatory mediator in different inflammatory autoimmune diseases, such as systemic lupus erythematosus (SLE) and ankylosing spondylitis (AS). Studies indicated that expression of plasma TL1A was elevated in SLE patients and was positively correlated with disease activity score (**Table 1**) (51). In a meta-analysis, the authors found that TL1A gene polymorphisms (rs3810936, rs7848647) may associate with SLE susceptibility (**Table 2**) (88). There are few studies about TL1A and SLE, and more evidence is needed to elucidate the role of TL1A in SLE pathogenesis. In addition, TL1A expression was upregulated in AS patients (**Table 1**) and TL1A gene rs3810936 TT genotype was associated with risk of AS in Chinese Han population (**Table 2**) (89, 90).

## Conclusion

Twenty years after discovery of TL1A, limited evidence has known about the role of TL1A. Based on available evidence, TL1A regulates homeostasis and inflammation *in vivo* through the TL1A/DR3/DcR3 pathway. For immune cells, TL1A promotes the proliferation and differentiation of various helper T cells and regulates the function of regulatory T cells. In inflammatory autoimmune diseases, TL1A expression was abnormal and correlated with disease activity, suggesting that TL1A is involved in the pathogenesis of the diseases. Genome-wide association studies (GWAS) showed that TL1A is associated with susceptibility to multiple inflammatory diseases, which may be an ideal therapeutic target. Nevertheless, what is the clear mechanism TL1A involved in the diseases? Whether targeting TL1A/DR3/DcR3 pathway is effective in inhibiting the diseases? There are some issues of concern yet. First, relationship between TL1A and B cells is rarely discussed, and it is still worth discussing whether TL1A can participate in the diseases development by regulating B cell function. Why? B cells are important adaptive immune cells that are able to produce various antibodies, especially autoantibodies in inflammatory autoimmune diseases. A second question is the potential of TL1A as a disease biomarker. In fact, more rigorous designs are needed to test the hypothesis, for example, a specific cohort of patients with preclinical, early and long-term diseases is recruited, in which expression of TL1A in the diseases patients has been proved to have diagnostic or predictive utility. Third, available studies showed that anti-TL1A antibody significantly improved the symptoms of IBD as discussed above, however, is it feasible to be used in other ethnic populations and to treat other inflammatory autoimmune diseases? Although much remains to be explored regarding the role of TL1A in the pathogenesis of autoimmune diseases, there is undeniable growing evidence supporting the significance of TL1A in autoimmune inflammatory diseases now. TNF-α and IL-6 were first discussed in large samples of different autoimmune diseases to evaluate the potential of them as disease markers. Then, functional studies regarding TNF-α and IL-6 were conducted to develop novel, small molecule targeted drugs for clinical treatment. Similarly, it is reported that anti-TL1A targeting IBD drugs have been used in phase III clinical trials. In our opinion, more multi-center, large population-based epidemiological studies in the future are still needed to explore the potential of TL1A as a disease marker for autoimmune diseases, especially in SLE. Furthermore, in-depth functional studies are urgent to develop TL1A small-molecule targeted drugs, providing evidence for the future clinical treatment of autoimmune diseases such as RA and SLE.

## Author Contributions

Study conception and design: W-DX, RL, A-FH. Acquisition of data: W-DX, RL, A-FH. Drafting the article or revising it: W-DX, RL, A-FH. Final approval of the version of the article to be published: all authors. All authors contributed to the article and approved the submitted version.

## Funding

This work was supported by grants from the National Natural Science Foundation of China (81701606) and Sichuan Provincial Natural Science Foundation (2022NSFSC0697, 2022NSFSC0694).

## Conflict of Interest

The authors declare that the research was conducted in the absence of any commercial or financial relationships that could be construed as a potential conflict of interest.

## Publisher’s Note

All claims expressed in this article are solely those of the authors and do not necessarily represent those of their affiliated organizations, or those of the publisher, the editors and the reviewers. Any product that may be evaluated in this article, or claim that may be made by its manufacturer, is not guaranteed or endorsed by the publisher.
